# Efficacy of Non-Invasive Ventilation in Acute Coronary Syndrome Patients with Acute Systolic Heart Failure

**DOI:** 10.31083/j.rcm2309294

**Published:** 2022-09-05

**Authors:** Chao Qu, Qi Zhao, Wei Cao, Zhenguo Dai, Xing Luo, Ruoxi Zhang

**Affiliations:** ^1^Department of Cardiology, Heilongjiang Provincial People's Hospital, 150036 Harbin, Heilongjiang, China; ^2^Department of Cardiology, 1st Affiliated Hospital of Harbin Medical University, 150001 Harbin, Heilongjiang, China; ^3^Department of Cardiology, Harbin Yinghua Hospital, 150119 Harbin, Heilongjiang, China; ^4^Department of Cardiology, 2nd Affiliated Hospital of Harbin Medical University, 150086 Harbin, Heilongjiang, China

**Keywords:** non-invasive ventilation, acute systolic heart failure, pulmonary edema, mortality, acute myocardial infarction, acute coronary syndrome, echocardiogram, dyspnea, hospitalization

## Abstract

**Background:**

Acute systolic heart failure (ASHF) is one of the most serious complications of the acute coronary syndrome (ACS), and increases the likelihood of adverse clinical outcomes. It remains unclear whether the use of non-invasive ventilation (NIV) could improve symptoms and reduce mortality in patients with ASHF derived from ACS.

**Methods:**

Data on biological, clinical, and demographic factors, as well as therapy data, were collected from patients with ASHF in the cardiac department. A total of 1257 ACS patients with ASHF were included in the study. Patients were divided into two groups. The control group received standard oxygen therapy. The comparison group consisted of those who underwent NIV as part of their immediate care. During hospitalization and at follow-up, information on both groups was systematically compared.

**Results:**

In comparison with the control group, mean 24-hour urine output was found to be significantly higher in the NIV group. A significant reduction in the duration of symptoms was observed among patients in the NIV group from the time of admission until relief of dyspnea. Heart rate, C-reactive protein, estimated glomerular filtration rate, and N-terminal prohormone of brain natriuretic peptide (NT-proBNP) was also improved, compared with those in the control group. The NIV group was found to have a higher survival rate. NIV was independently related to all-cause mortality in 1-year follow-up (hazard ratio, 0.674; *p* = 0.045).

**Conclusions:**

Our study shows that NIV, as compared with standard oxygen therapy, has a beneficial impact on heart rate, metabolic balance, and relief of dyspnea in ACS patients with ASHF which results in reduced intubation rate, duration of in-hospital stay, and 1-year mortality.

## 1. Introduction

Acute coronary syndrome (ACS) with acute systolic heart failure (ASHF) is a common medical condition that may be present when the patient is admitted to the hospital or may develop during hospitalization. In patients with ACS, the degree of heart failure is closely related to mortality [1]. Clinically speaking, signs and symptoms related to systemic congestion such as accumulation of extracellular fluid (initiated by increased biventricular cardiac filling pressures) are primarily observed in ASHF [2, 3]. In patients with ASHF, adverse cellular and anatomic changes can occur, promoting disease progression and adverse clinical outcomes as a result of abnormal loading conditions and enhanced ventricular wall stress, which is associated with 90-day readmission rates and 1-year mortality (10–30%) [4, 5].

Initial treatment in most patients with ASHF involves non-invasive ventilation and intravenous diuretics, which are either administered alone (typically in Europe and Asia) or combined with short-acting vasodilators [6]. A large number of studies focused on the impact of non-invasive ventilation on the prognosis of patients with acute heart failure has yielded inconsistent results [7, 8, 9]. There is limited evidence that non-invasive ventilation improves short- and long-term outcomes in ACS patients with ASHF. Therefore, we conducted this study focusing on evaluating our experience with non-invasive ventilation (NIV) in ASHF patients.

## 2. Materials and Methods

### 2.1 Study Population

**Fig. 1.**
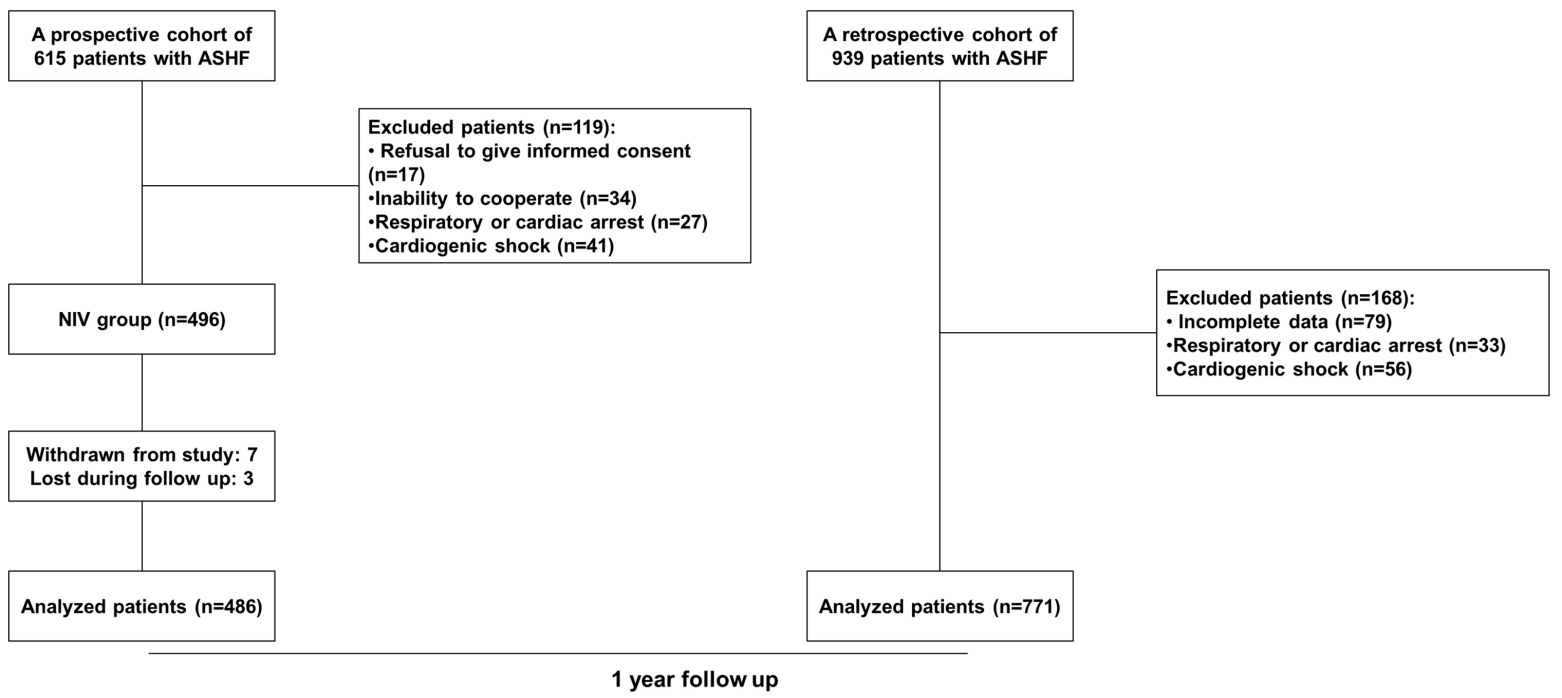
**The study flow diagram**. ASHF, acute systolic heart failure; NIV, non-invasive ventilation.

In total, 1257 participants were enrolled. Patients were divided into two groups based on the period of admission, from September 2010 to April 2014 and from September 2020 to March 2022 (Fig. 1). All patients had no prior history or clinical symptoms of acute heart failure (AHF) and were diagnosed with ACS with ASHF during hospitalization. The first group was a prospective group of 486 ACS patients with ASHF who underwent non-invasive mechanical ventilation. The control group was a retrospective group of 771 ACS patients with ASHF, receiving standard oxygen therapy alone prior to the introduction of non-invasive ventilation in our department after April 2014.

ACS with ASHF refers to new signs and symptoms of failure of the heart’s systolic function, featuring reduced ejection fraction and ventricular dilation, and is a major cause of unplanned hospital admission [10, 11]. Study participants were allowed to enroll if they were over 18 years old and hospitalized due to dyspnea at rest with at least one of the following three conditions: raised jugular venous pressure, peripheral edema, or pulmonary congestion [12]. The exclusion criteria were as follows: informed consent refused, cooperative inability, admission with cardiac or respiratory arrest, patients requiring emergency intubation, patients in shock on admission who needs for vasoactive drugs.

### 2.2 Data Collection

Clinical status at baseline and at discharge was classified in accordance with the New York Heart Association (NYHA) standards. Basic vital signs monitoring (blood pressure, body temperature, oxygen saturation, and heart rate), and production of urine, and levels of blood lactate were measured. From the initial day of hospitalization, we documented the following clinical features of the patient: New York Heart Association functional class, systemic arterial pressure, heart rate and echocardiographic. Laboratory data: oxygen saturation of arterial blood (${\mathrm{SaO}}_{2}$), 24-hour urine output, white blood cell count, serum creatinine, C-reactive protein (CRP), N-terminal B-type natriuretic peptide (NT-proBNP), troponin I and serum sodium Glomerular filtration rate on admission to the hospital was estimated using the simplified modification of diet in renal disease (MDRD) algorithm: estimated glomerular filtration rate (eGFR) = 186.3 (serum creatinine)^–1.154^ (age)^–0.203^ (female: ×0.742) [13].

### 2.3 Echocardiography

Echocardiography was performed in all ACS patients with ASHF symptoms. Expert sonographers performed echocardiograms with a 2.5-MHz transducer using Vivid 5 ultrasound equipment (GE Healthcare, Horten, Norway). Another experienced investigator conducted offline analysis using commercially available software (EchoPac, version 8; GE Healthcare, Horten, Norway) for all examinations. According to the European Association of Cardiovascular Imaging and the American Society of Echocardiography [14], the echocardiogram is operated by a double-blinded technologist.

### 2.4 Treatment

In the cardiology department, NIV was performed according to standardized procedures. Using a Respironics Synchrony ventilator, patients were administered continuous positive airway pressure (CPAP) with either a full-face mask or a total-face mask as the first course of treatment [15]. An initial positive end-expiratory pressure of 4–10 cm $H_{2}$O was observed, adjusted to improving patient comfort. As soon as symptoms of dyspnea were present after 30 minutes of CPAP treatment, NIV was switched to non-invasive intermittent positive pressure ventilation (NIPPV). To maintain peripheral oxygen saturation above 95%, the patient received up to 15 liters of supplemental oxygen every minute, with a maximum fraction of inspired oxygen of 0.6.

### 2.5 Clinical Outcomes and Definition

All patients were followed up 1 year after discharge in either an outpatient clinic or via telephone. A prior study defined all-cause death as death from any cause, and rehospitalization for heart failure as clinically diagnosed acute heart failure requiring hospitalization for >72 hours along with treatment by the administration of intravenous diuretics [16, 17, 18].

### 2.6 Statistical Analysis

In continuous data, the mean value is expressed as a percentage, while in categorical data, the frequency is expressed. Comparisons between groups were conducted using an independent two-sample *t*-test. Categorical variables were compared using either the Chi-square test or Fisher’s exact test, if applicable. Cox proportional hazards models were used to assess the relationship between patient characteristics and all-cause mortality. All baseline characteristics with *p *< 0.10 on univariate analysis were included in the multivariate models. Estimation of survival status was achieved by Kaplan-Meier (KM) technique and comparison of survival distributions was done by log-rank test. Statistical significance was set at two-sided *p*-values of <0.05. All statistical analyses were carried out via using SPSS version 22.0 (SPSS Inc., Chicago, IL, USA).

## 3. Results

### 3.1 Baseline Characteristics

The screening process involved 1554 patients. Among them, 1257 were finally enrolled in the study from September 2010 to March 2022. There was no significant difference in the characteristics of the two group’s baseline demographics and angiography (Table 1).

**Table 1.** **Baseline characteristics of patients at admission with acute systolic heart failure**.

		NIV group (n = 486)	Control group (n = 771)	*p*-value
Age, years		66.18 ± 9.74	66.38 ± 9.57	0.725
Men, n (%)		335 (68.93)	551 (71.47)	0.342
Co-existing conditions, n (%)				
	Hypertension	285 (58.64)	440 (57.07)	0.598
	Diabetes mellitus	160 (32.92)	230 (29.83)	0.260
	Smoker	163 (33.54)	249 (32.30)	0.666
Vital signs				
	Systolic blood pressure, mmHg	161.14 ± 19.31	159.93 ± 19.69	0.282
	Heart rate, beats/min	110.50 ± 10.24	110.22 ± 9.89	0.628
	Respiratory rate, breaths/min	25.15 ± 3.99	24.84 ± 3.97	0.179
	Cardiogenic pulmonary edema	117 (24.07)	193 (25.03)	0.737
Medication prior to admission, n (%)				
	Beta blockers	291 (59.88)	444 (57.59)	0.445
	Spironolactone	281 (57.82)	429 (55.64)	0.483
	Diuretics	241 (49.59)	368 (47.73)	0.524
	ACEi/ARB	288 (59.26)	448 (58.11)	0.724
Laboratory testing				
	WBC, ×${\mathrm{10}}^{9}$/L	12.70 ± 3.53	13.00 ± 3.90	0.173
	CRP, mg/L	25.49 ± 9.13	24.84 ± 8.71	0.211
	NT-proBNP, pg/mL	5101.12 ± 2427.85	5068.22 ± 2381.83	0.814
	Troponin I, μg/L	14.78 ± 6.54	15.23 ± 6.67	0.240
	eGFR, mL/min/1.73 $m^{2}$	55.00 ± 13.88	55.18 ± 14.11	0.827
Initial blood gas analysis				
	${\mathrm{SaO}}_{2}$, %	80.88 ± 2.81	81.02 ± 2.89	0.384
	${\mathrm{PaO}}_{2}$, mmHg	63.45 ± 2.51	63.49 ± 2.45	0.764
	${\mathrm{PaCO}}_{2}$, mmHg	62.24 ± 3.83	61.86 ± 4.07	0.095
	Lactic acid, mmol/L	4.07 ± 1.78	4.13 ± 1.73	0.551
Echocardiographic parameters				
	LVEDD, mm	57.07 ± 3.89	57.16 ± 3.93	0.675
	LVEF, %	42.98 ± 2.94	42.90 ± 3.16	0.681

Mean values (standard deviation) and % (n) were reported for continuous and categorical variables, respectively. NIV, non-invasive ventilation; ACEi, angiotensin-converting enzyme inhibitor; ARB, angiotensin receptor blocker; WBC, white blood cell; CRP, C-reactive protein; NT-proBNP, N-terminal pro-B type natriuretic peptide; eGFR, estimated glomerular filtration rate; ${\mathrm{SaO}}_{2}$, oxygen saturation of arterial blood; ${\mathrm{PaO}}_{2}$, alveolar oxygen partial pressure; ${\mathrm{PaCO}}_{2}$, partial pressure of carbon dioxide in artery; LVEDD, left ventricular end diastolic diameter; LVEF, left ventricular ejection fraction.

### 3.2 Patients’ Characteristics at Discharge

The patients’ characteristics at discharge are illustrated in Table 2. In the NIV group, mean 24-hour urine output was considerably higher than in the control group. For patients in the NIV group, duration from admission to dyspnea relief, heart rate, C-reactive protein (CRP), estimated glomerular filtration rate (eGFR), and NT-proBNP were significantly lower than for those in the control group.

**Table 2.** **Comparison of patients’ characteristics at discharge**.

		NIV group (n = 479)	Control group (n = 741)	*p*-value
Drugs in hospital, n (%)				
	Nitroprusside/nitrates	437 (91.23)	678 (87.94)	1.000
	Levosimendan			
	Loop diuretics	471 (98.33)	728 (98.25)	1.000
Vital signs				
	Systolic blood pressure, mmHg	118.11 ± 5.27	118.12 ± 4.95	0.971
	Heart rate, beats/min	84.89 ± 3.96	88.23 ± 6.75	<0.001
	Respiratory rate, breaths/min	19.14 ± 2.04	19.01 ± 2.08	0.295
Laboratory testing				
	WBC, ×${\mathrm{10}}^{9}$/L	7.89 ± 1.91	7.96 ± 1.98	0.543
	CRP, mg/L	8.02 ± 2.88	10.05 ± 3.77	<0.001
	eGFR, mL/min/1.73 $m^{2}$	54.60 ± 14.00	51.65 ± 11.95	<0.001
	NT-proBNP, pg/mL	437.05 ± 178.80	722.88 ± 332.37	<0.001
	Troponin I, μg/L	1.24 ± 0.18	1.23 ± 0.19	0.336
	Mean 24-h urine output, mL/day	1284.35 ± 232.89	1102.99 ± 189.22	<0.001
Blood gas analysis				
	${\mathrm{SaO}}_{2}$, %	94.06 ± 1.03	94.05 ± 1.03	0.932
	${\mathrm{PaO}}_{2}$, mmHg	80.06 ± 2.05	79.93 ± 1.98	0.279
	${\mathrm{PaCO}}_{2}$, mmHg	44.10 ± 2.06	44.02 ± 1.88	0.482
	Lactic acid, mmol/L	1.61 ± 0.21	1.61 ± 0.20	0.710
Echocardiographic parameters				
	LVEDD, mm	55.17 ± 2.89	55.06 ± 2.87	0.526
	LVEF, %	48.05 ± 3.03	48.01 ± 3.09	0.839

Mean values (standard deviation) and % (n) were reported for continuous and categorical variables, respectively. NIV, non-invasive ventilation; WBC, white blood cell; CRP, C-reactive protein; NT-proBNP, N-terminal pro-B type natriuretic peptide; eGFR, estimated glomerular filtration rate; ${\mathrm{SaO}}_{2}$, oxygen saturation of arterial blood; ${\mathrm{PaO}}_{2}$, alveolar oxygen partial pressure; ${\mathrm{PaCO}}_{2}$, partial pressure of carbon dioxide in artery; LVEDD, left ventricular end diastolic diameter; LVEF, left ventricular ejection fraction.

### 3.3 Clinical Events during Hospitalization and 1 Year Follow up

**Fig. 2.**
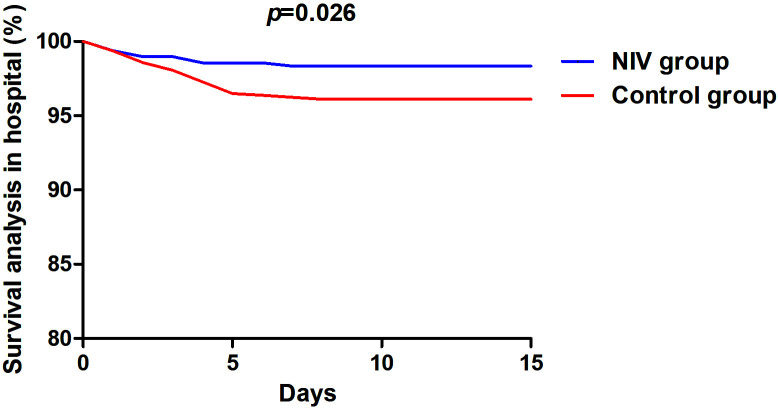
**Survival analysis in hospital**. NIV, non-invasive ventilation.

**Fig. 3.**
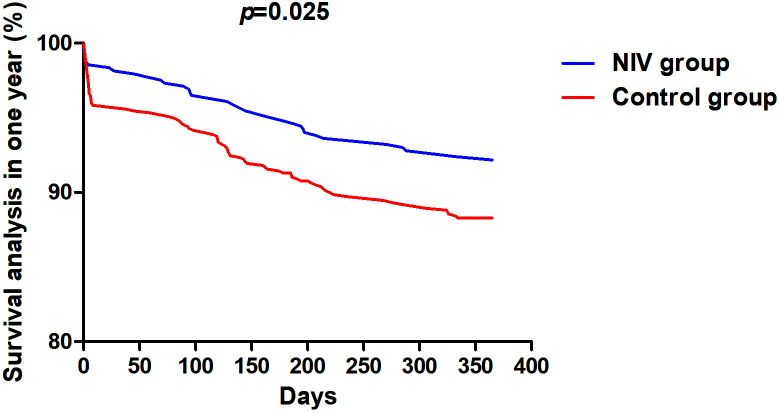
**Survival analysis in 1-year follow up**. NIV, non-invasive ventilation.

Cardiac death rate, rate of intubation, and length of hospital stay were significantly lower in the NIV group than in the control group (1.33% vs. 3.89%, *p* = 0.027, 3.50% vs. 6.36%, *p* = 0.027 and 10.01 ± 2.12 days vs. 10.01 ± 2.12 days, *p *< 0.001; Table 3 and Fig. 2) during hospitalization. All-cause mortality (7.61% vs. 11.93%, *p* = 0.017), cardiac death (5.56% vs. 10.51%, *p* = 0.003), and rate of NIV treatment in rehospitalization differ significantly between the two groups in the 1-year follow-up (Table 3 and Fig. 3).

**Table 3.** **In-hospital and 1-year events**.

		NIV group (n = 486)	Control group (n = 771)	*p*-value
In-hospital				
	Cardiac death, n (%)	7 (1.44)	30 (3.89)	0.027
	Intubation, n (%)	17 (3.50)	49 (6.36)	0.027
	Length of hospital stay (days)	10.01 ± 2.12	14.07 ± 1.99	<0.001
1-year				
	All-cause mortality, n (%)	37 (7.61)	92 (11.93)	0.017
	Cardic death	27 (5.56)	81 (10.51)	0.003
	Rehospitalization associated with heart failure, n (%)	274 (56.38)	408 (52.92)	0.245
	NIV treatment in rehospitalization, n (%)	201 (41.35)	0 (0.00)	<0.001

Mean values (standard deviation) and % (n) were reported for continuous and categorical variables, respectively. NIV, non-invasive ventilation.

Univariate Cox regression analysis indicated that NIV (hazard ratio = 0.651, *p* = 0.026), CRP (hazard ratio = 1.198, *p *< 0.001), and NT-proBNP (hazard ratio = 1.001, *p *< 0.001) were associated with 1-year all-cause mortality (Table 4). In addition, multivariate Cox regression analysis showed that NIV (hazard ratio = 0.674; *p* = 0.045) and NT-proBNP (hazard ratio = 1.001, *p *< 0.001) were independently associated with 1-year all-cause mortality (Table 4).

**Table 4.** **Univariate and multivariate cox regression analysis for all-cause mortality in one year**.

	Univariate		Multivariate	
	Hazard ratio (95% CI)	*p*-value	Hazard ratio (95% CI)	*p*-value
NIV	0.651 (0.445–0.950)	0.026	0.674 (0.458–0.991)	0.045
Heart rate	1.007 (0.989–1.024)	0.460	-	-
CRP	1.198 (1.178–1.218)	<0.001	0.946 (0.789–1.134)	0.550
eGFR	0.995 (0.983–1.007)	0.432	-	-
NT-proBNP	1.001 (1.000–1.002)	<0.001	1.001 (1.000–1.002)	0.004

CI, confidence interval; NIV, non-invasive ventilation; CRP, C-reactive protein; NT-proBNP, N-terminal pro-B type natriuretic peptide; eGFR, estimated glomerular filtration rate.

## 4. Discussion

The major findings of this study are as follows: (1) NIV significantly reduced cardiac death and hospitalization intubation rates as well as the length of hospital stay in ACS patients with ASHF as compared with the control group. (2) At 1-year follow-up, all-cause mortality and cardiac death rate were significantly lower in the NIV group than in the control group. (3) The Cox model showed that not using NIV was an independent risk factor for all-cause mortality in ACS patients with ASHF. These results suggest that NIV treatment has a positive effect on the prognosis of ACS patients with ASHF.

In ASHF, patients with acute cardiogenic pulmonary edema tend to experience significant respiratory failure. The use of NIV, which provides positive intrathoracic pressure through an interface, is effective in treating some moderate to severe respiratory failure cases [19]. It has been reported that NIV use is associated with a lower incidence of endotracheal intubation in ASHF patients with ischemic etiology [20]. Furthermore, Zhu *et al*. [21] also reported that, in patients undergoing cardiothoracic surgery, the use of NIV improved patient’s oxygenation and decreased the need for endotracheal intubation. In our study, we also found that NIV groups showed lower endotracheal intubation rates. In addition, we also observed a significant reduction in mortality in the NIV group, both in hospital and at 1-year follow-up. However, another study, which included patients with ischemic cardiomyopathy and chronic obstructive pulmonary disease, found that NIV did not significantly improve short-term outcomes in patients with AHF [20]. In our study, which found positive improvements with NIV, we only enrolled ACS patients with ASHF. Another study, by Tanaka *et al*. [22], supports our view by reporting that the vital signs and oxygenation was improved in pulmonary edema patients received NIV treatment, and the intubation rate was also decreases. Evidence suggests that NIV can help improve blood oxygen content and hemorheological status, as well as minimize plasma lipid peroxidation injury [23]. Furthermore, our study suggests that the NIV can not only improve oxygen saturation in ACS patients with ASHF but also improve their survival rate. A previous study also demonstrated that continuous positive airway pressure treatment significantly reduced cognitive defects associated with obstructive sleep apnea, which decreased the incidence of major adverse cardiac events [24]. The cognitive level of patients was not evaluated in the present study, which is a limitation of this study.

**Fig. 4.**
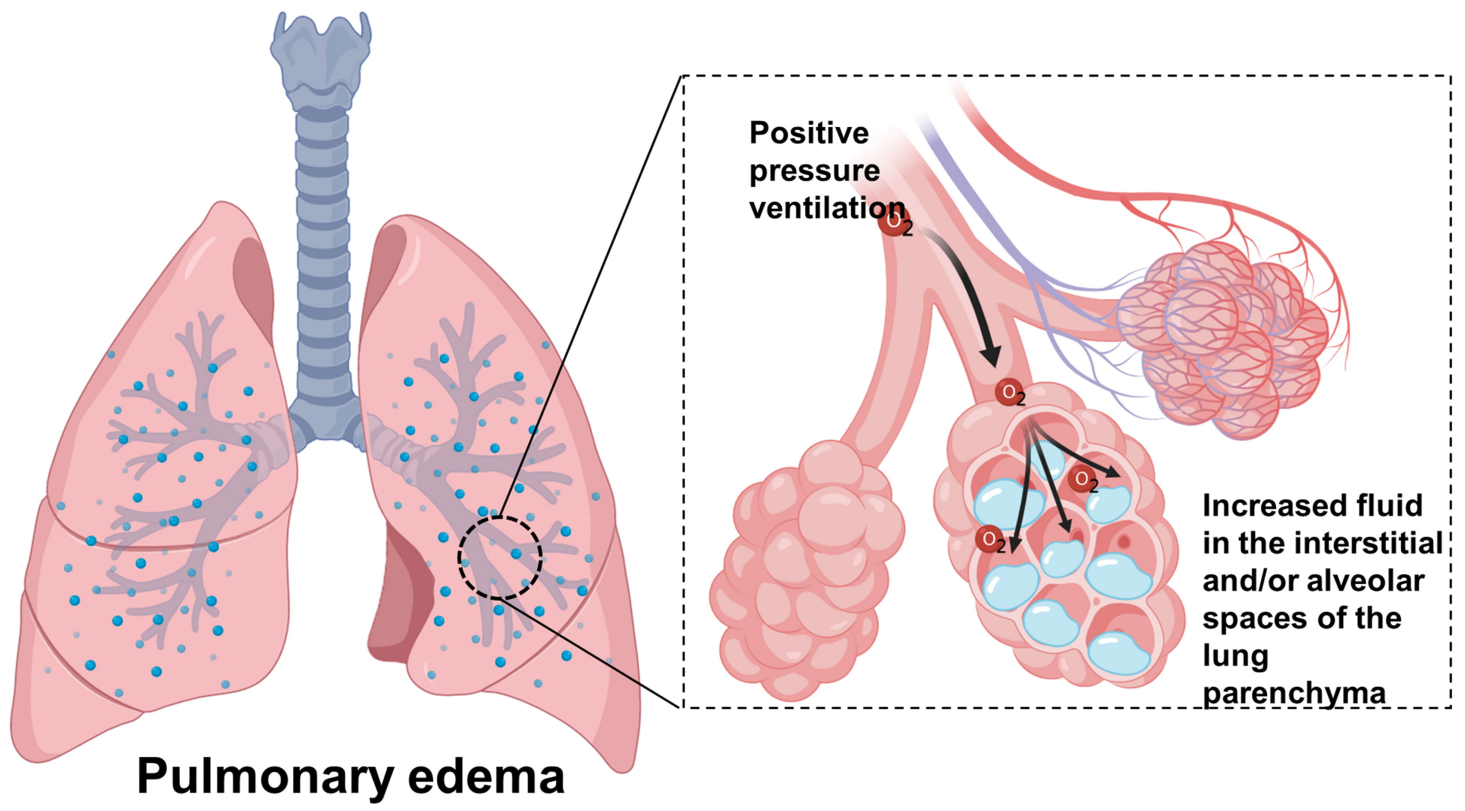
**Positive pressure ventilation and cardiogenic pulmonary edema**.

The second most common indication for NIV is acute cardiogenic pulmonary edema, secondary to ASHF [25]. In patients with cardiogenic pulmonary edema who received NIV treatment, CPAP can rapidly improve oxygenation by re-expanding flooded alveoli and increase functional residual capacity, thereby positioning the lung on its compliance curve in a more favorable way (Fig. 4), which results in a reduction in breathing difficulty and improved cardiac performance [26]. The latter may be achieved by raising pericardial pressure, lowering left ventricular transmural pressure (systolic wall stress), and subsequently reducing afterload [27]. In our study, the efficacy of NIV in rapidly improving dyspnea, vital signs, and metabolic balance was established in ACS patients with ASHF. NIV resulted in significant improvements in heart rate, mean 24-hour urine output, CRP, eGFR, and NT-proBNP.

ASHF is characterized by an increased heart rate, which determines the amount of oxygen consumed and energy burnt by the myocardium [28]. Increased heart rate modulates cardiac afterload, reduces diastolic perfusion time, and triggers ischemic events and arrhythmias. A higher heart rate is often observed in patients with ASHF, which can be a compensatory mechanism to improve cardiac output as a result of vasopressor amines or as a contributing factor to clinical deterioration. Therefore, the reduction of heart rate may be among the most effective ways to save energy in patients with cardiogenic shock and multiorgan failure [29]. In patients who are hospitalized for ASHF, inflammation is also common. CRP, an acute-phase reactant that can be used to evaluate systemic inflammation, represents a nonspecific marker of inflammation [30]. Interestingly, numerous previous studies have reported that elevated CRP levels in the hospital may correlate with poor prognosis in patients with ASHF [31]. In our research, the CRP levels at discharge of patients in the NIV group were lower than of those in the control group, indicating that NIV treatment may have improved the clinical prognosis to a certain extent.

Patients presenting with ASHF have been reported to be affected by adverse clinical outcomes such as the possibility of intubation, stroke, malignant arrhythmia, rehospitalization, and unexpected demise [32]. According to the current study, the risk of endotracheal intubation is reduced by nearly half with NIV in comparison with standard therapy, which is in line with the majority of previous studies investigating the benefits of NIV, verifying the success and appropriateness of our therapeutic intervention [8]. Meta-analysis and systematic reviews have found that patients with acute cardiogenic pulmonary edema who were treated immediately with noninvasive ventilation had a 47% reduction in mortality [33, 34]. A similar rate of cardiac mortality was reported in our study compared to that reported by registries for patients with acute heart failure (6.7% in the EuroHeart Failure Survey II and 4% in the Acute Decompensated Heart Failure National Registry [ADHERE]) [35, 36].

To our surprise, our data demonstrated significant improvements in haemodynamic parameters, diuresis, and biochemical indices in the treated subjects, without observed differences in echocardiographic parameters. There are two possible reasons for this result. Firstly, a larger number of patients died in the control group (30 vs. 7) and so were excluded from the group’s echocardiographic parameters as these were assessed at discharge. This maybe contribute to absence of significant differences in echocardiographic parameters between the two groups due to all survivors having improved characteristics. Secondly, cardiac ultrasound is used to evaluate structural changes and is less sensitive than hemodynamic changes [37]. The patients in this study, all of whom had post-ischemic heart failure, were in the necrotic and edematous phases of cardiac myocytes, and no fibrotic repair had occurred. Therefore, changes in ejection fraction as well as cardiac structure had not yet occurred.

Our study has some limitations. First, it was a single-center study, and treatment choices were made based on physician preference, introducing a natural bias. However, the staff at the department of cardiology were competent and experienced in terms of the use of NIV in patients with ASHF. Second, learning bias could not be avoided due to this study not being a randomized controlled trial. Third, some of the patients who participated in our study received beta-blockers and so it was impossible to draw firm conclusions about acute changes in heart rate from our group.

## 5. Conclusions

In patients with ASHF, improvement occurs more rapidly in dyspnea with better effect on heart rate and metabolic balance with NIV than with standard oxygen therapy, effectively reducing intubation rate, duration of hospital stays, and 1-year mortality. To validate these findings, further large randomized controlled trials involving larger numbers of patients will be required.

## Data Availability

The raw data supporting the conclusions of this article will be made available by the authors, without undue reservation.
